# Supplementary material for: Terahertz photonic heterodyne spectral analysis with (sub-) kHz resolution and 6.5 THz frequency coverage
Source: Nat Commun. 2025 Nov 25;16:10594. doi: 10.1038/s41467-025-66457-6 (PMC12658009; doi:10.1038/s41467-025-66457-6)
Supplement: Supplementary file 1 — Supplementary Information [file 41467_2025_66457_MOESM1_ESM.pdf]

Supplemental to “Terahertz photonic heterodyne spectral analysis with (sub-) kHz resolution and 6.5 THz frequency coverage”

CALIBRATION

For the calibration of the heterodyne measurements, we employ the responsivity (see eq. (6)) acquired from homodyne measurements with the employed Rh:InGaAs photoconductive receiver. The homodyne system employs the same local oscillator signal for both the transmitter and receiver. The transmitter is a waveguide-integrated photodiode provided by the Fraunhofer Heinrich-Hertz-Institute (HHI) and the local oscillator consists of a Keysight 81960A at 1540 nm, along with a tunable Keysight 81608A at longer wavelengths. Their difference frequency is the local oscillator frequency. Both the transmitter and the receiver are driven by an optical power of 30 mW each. A lock-in amplifier modulates the transmitter bias with a sinusoidal shape that also provides the reference for demodulation of the received signal. This measurement determines the photocurrent I_R of the photoconductive receiver in response to a given Terahertz electrical field on the input side. Following the responsivity equation (Eq. 6 in the methods section), the photocurrent is squared and the power of the calibration source determined by a gauged pyroelectric detector (SLT Sensor- und Messtechnik THz10). This pyroelectric detector is calibrated at a frequency of 1.4 THz at the German National Metrology Institute PTB and possesses a noise floor of 1 µW. The power of the photodiode decreases for higher THz frequencies due to roll-off effects, limiting the measurable frequency range to 1.6 THz. We assume a maximum error of 30 % in responsivity of the pyroelectric detector due to the difference between its calibration frequency and the range of frequencies used in the homodyne measurements. The blue curve in Fig. S1 shows the determined responsivities of the Rh:InGaAs photoconductive mixer for the frequency range up to 1.6 THz. As we measured signals up to frequencies of 6.5 THz, we extrapolated the responsivity for frequencies higher than 1.6 THz (orange line). The extrapolation is based on the known roll-off effects present in the receiver.

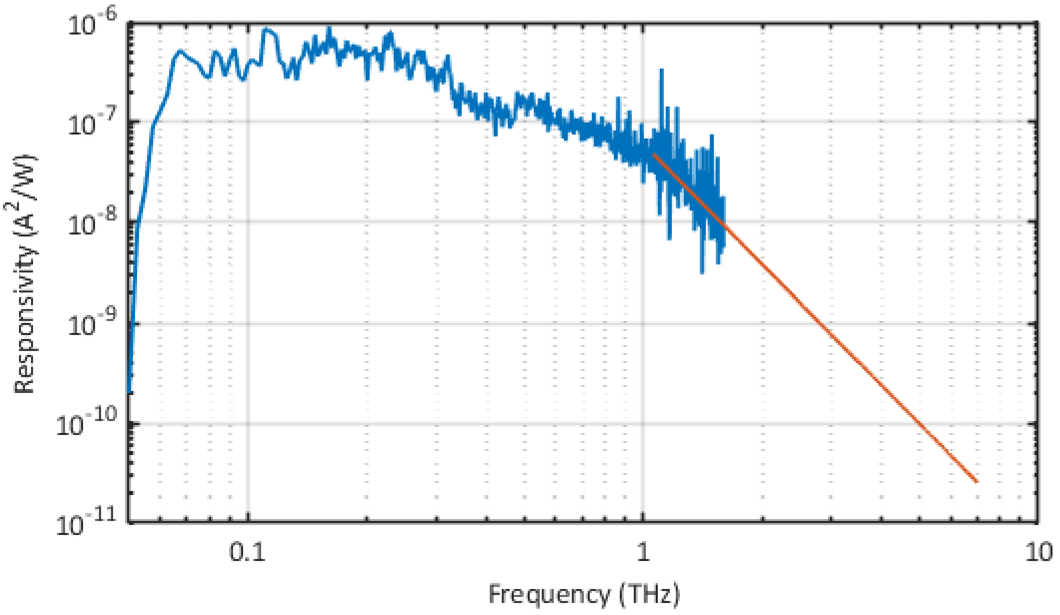

Fig. S1: Measured responsivity of the Rh:InGaAs photoconductive receiver for a frequency range from 50 GHz to 1.6 THz (blue line) with an extrapolated estimation based on a f^{-4} approximation for the frequency range above 1.1 THz until 7 THz (orange line).

For the photoconductor, the lifetime 3dB roll-off frequency is approximately 600 GHz while the RC 3 dB frequency is approximately at 1.06 THz. For any higher frequency the responsivity will fall approximately with f^{-4}. The measurements for frequencies between 1.06 THz and 1.6 THz confirm this trend. It should be noted that the antenna performance (radiation resistance R_A) will become frequency-dependent beyond 2-3 THz, affecting both the RC roll-off as well as the power coupled by the antenna as $\mathcal{R} \sim \frac{\frac{1}{2} R_A I_{THz}^2}{1+(2\pi R_A C f)^2} \sim \frac{1}{R_A}$ at high frequencies. As a result, we must conservatively assume a maximum additional error of 5 dB for the extrapolation to 6.5 THz.

The calibration used a different local oscillator than the heterodyne measurements of the main manuscript but both local oscillators use a measured optical power of 30 mW.

We confirmed the accuracy of the responsivity calibration with the spectral and power measurement of a TOPTICA Terascan at a frequency of 95 GHz. A spectral measurement with a spectrum analyzer measures the power in each frequency bin for the corresponding resolution bandwidth. Integrating over the frequency spectrum equals the power of the signal. To confirm, we measure the power separately, calculate the responsivity from the spectrum and compare it to the responsivity acquired with the homodyne measurements. The difference between both calibration techniques is 1.4 dB which is well within the estimated errors of the calibration.

The calculation of the displayed average noise level uses the responsivity, the noise floor of the spectral measurements of the T-Sweeper and the resolution bandwidth. The current noise floor of the photoconductive mixer remained constant throughout the measurements, independent of the local oscillator frequency at constant optical power, and the power noise floor scales linearly with the resolution bandwidth.

SPECTROSCOPY

In the section ‘homodyne CW-THz-spectroscopy’ we introduced the spectroscopy system. Besides the low pressure gas spectroscopy measurement in the main manuscript, we also used the homodyne setup to determine the reflectance of a Silicon (Si) wafer with a thickness of 380 µm. In combination with the air surrounding it, the Si wafer forms a Fabry-Pérot resonator which only transmits specific frequencies efficiently. Accordingly, broad parts of the spectrum will get reflected as Fig. S2 proves. Positive reflectance values stem from frequency points at which the reference (Fig. 7b) and the reflection sample measurement are limited either by the noise floor of the device or by the absorption from the water vapor present in both measurements. The measurement in Fig. S2 demonstrates thus another high resolution application example.

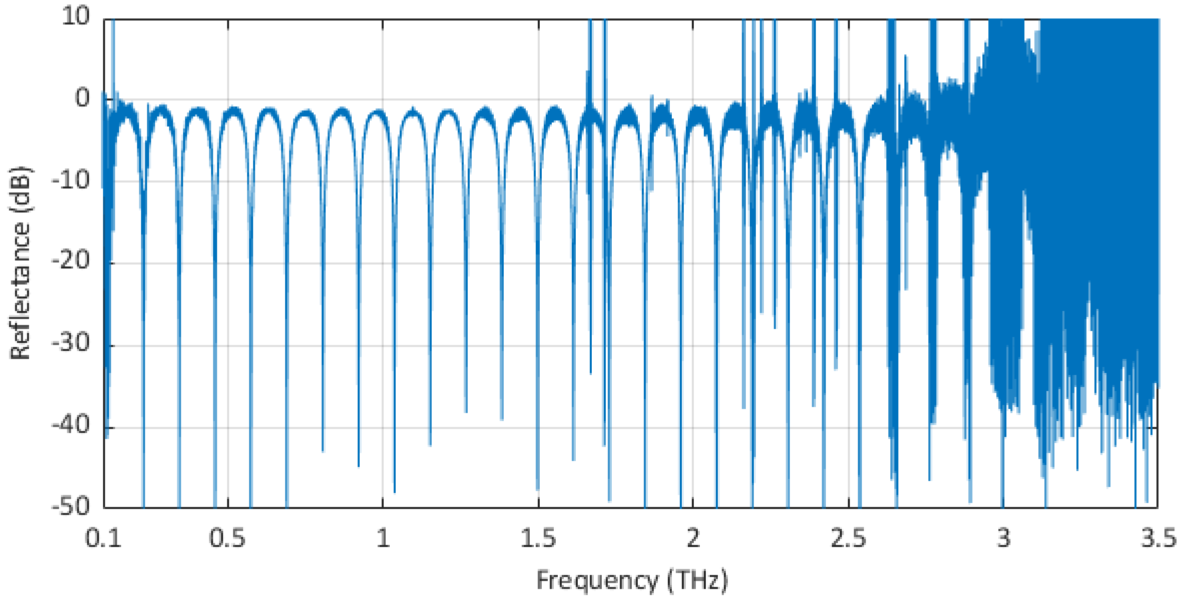

Fig. S2: Acquired reflectance of a Si wafer with a thickness of 380 µm. Determined from the reflected photocurrent measurement of the Si wafer and the reference signal of Fig. 7b).

WATER VAPOUR ABSORPTION SPECTROSCOPY

For the absorption spectroscopy we applied the LO to both the photoconductive transmitter and receiver (Fig. S3). These were positioned opposite each other, each with a TPX-lens for beam forming. In between the lenses we positioned an evacuated cell that can be filled with a gas under test at a desired pressure. The vacuum cell has two fused silica windows positioned under their Brewster angle to reduce reflections. Separated by a needle valve, we attached a probe volume to the vacuum cell where we deposited a drop of ammonia solution. The intention of using ammonia was to record both water and ammonia lines. On the other side the vacuum tube connects to a flooding vent, a pressure gauge and a valve to the pump. The pump is a combined unit of a membrane pump and a turbomolecular pump. During the measurements the pressures of the pressure gauge are logged. A bias of -1.2 V is applied to the photodiode transmitter. For the measurements the output of the receiver is amplified by $3.3 \cdot 10^5$ V/A by a TEM Messtechnik PDA-S transimpedance amplifier and acquired by a LeCroy HDO oscilloscope at 500 kSa/s. For the measurement the LO is swept 30 GHz up and down surrounding the water vapour absorption peak at a sweep rate of 11.8 GHz/s. Each measurement is the average of the Hilbert transformation of 3 up and down sweeps.

Before the start of the measurements we evacuated the cell to pressures below 0.01 mbar and partially the probe chamber to remove remaining air. We also flood the THz path outside the chamber with dry air to remove any water vapor potentially influencing the measurement. The finite gas pressure of water and ammonia filled the probe volume with an ammonia-water gas mixture. Care was taken to slowly flood the gas chamber with the ammonia-water gas mixture by slowly opening the needle valve with the pumps running and connected to the vacuum chamber. Except for the lowest pressures, we cut off the vacuum pumps after approximately 2 minutes and slowly let more probe into the test cell. The leak tightness of the vacuum cell is more in the order of 50 mbar over the course of 30 minutes, i.e. the pressure-broadened linewidths contain some foreign broadening by leakage oxygen and nitrogen into the chamber as well as minor concentrations of ammonia.

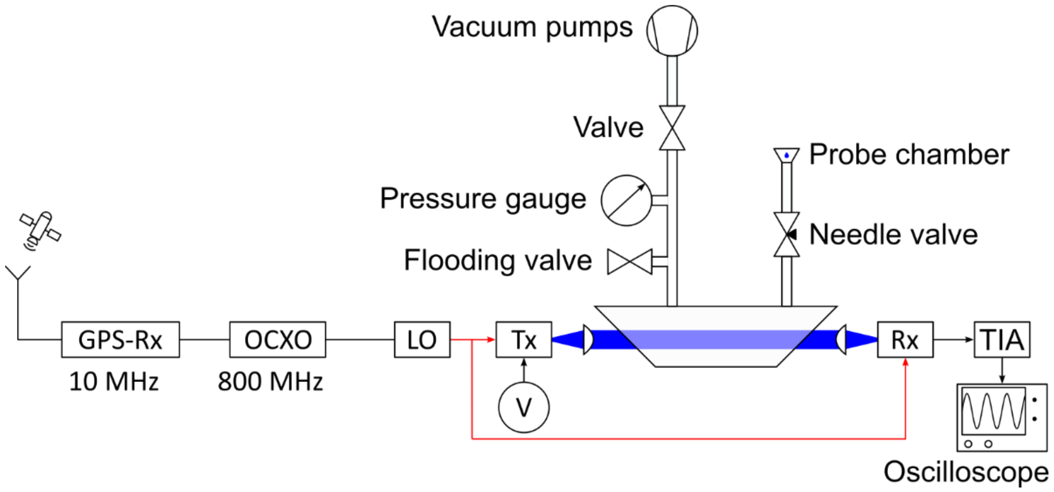

Fig. S3: Schematic of the heterodyne measurements with vacuum tube
